# Optimization of Tumor Targeting Gold Nanoparticles for Glioblastoma Applications

**DOI:** 10.3390/nano12213869

**Published:** 2022-11-02

**Authors:** Nicholas C. Allen, Rajat Chauhan, Paula J. Bates, Martin G. O’Toole

**Affiliations:** 1 Department of Bioengineering, University of Louisville, Louisville, KY 40292, USA; 2 Department of Medicine, University of Louisville School of Medicine, Louisville, KY 40202, USA

**Keywords:** glioblastoma, gold nanoparticles, AS1411

## Abstract

Glioblastoma brain tumors represent an aggressive form of gliomas that is hallmarked by being extremely invasive and aggressive due to intra and inter-tumoral heterogeneity. This complex tumor microenvironment makes even the newer advancements in glioblastoma treatment less effective long term. In developing newer treatment technologies against glioblastoma, one should tailor the treatment to the tumor microenvironment, thus allowing for a more robust and sustained anti-glioblastoma effect. Here, we present a novel gold nanoparticle therapy explicitly designed for bioactivity against glioblastoma representing U87MG cell lines. We employ standard conjugation techniques to create oligonucleotide-coated gold nanoparticles exhibiting strong anti-glioblastoma behavior and optimize their design to maximize bioactivity against glioblastoma. Resulting nanotherapies are therapy specific and show upwards of 75% inhibition in metabolic and proliferative activity with stark effects on cellular morphology. Ultimately, these gold nanotherapies are a good base for designing more multi-targeted approaches to fighting against glioblastoma.

## 1. Introduction

Glioblastomas (GBMs) are the most frequent primary brain tumors in adults accounting for approximately 57.3% of diagnosed brain gliomas [1]. Diagnoses have a median prognosis of 13.5 months [2] and a five-year survival rate of 22, 9, or 6% based on age ranges of 20–44, 45–54, and 55–64, respectively [3,4,5,6,7,8,9]. Management and outcome of GBM-affected patients have remained consistent for almost 40 years [2,10], with surgery being an essential component of GBM treatment; however, complete resection is not always available [11,12]. The most promising treatment of GBM is a combination therapy of radio and chemotherapies (such as temozolomide) [13,14,15]. Still, radio or chemotherapies have potential toxicity to the surrounding normal brain [12,15,16,17,18,19]. These therapies may contribute to hematological complications, cause fatigue, and are implicated in an increased rate of infections among patients [12,16,17,18]. Due to its aggressive nature, clinicians suggest that the development of new GBM treatments should utilize rational combinations of therapies aiming for the inhibition of angiogenesis, the induction of apoptosis, or the inhibition of several signal transduction pathways [13]. Fortunately, advances in understanding genetic mechanisms behind GBM formation and survival have allowed for the possible generation of such therapies.

New oligonucleotide-based therapies have been determined to be bioactive against GBM in vitro [20,21,22,23,24,25,26,27]. Among these is AS1411, a synthetic 26-nucleotide phosphodiester oligodeoxynucleotide with the sequence 5′-GGTGGTGGTGGTTGTGGTGGTGGTGG. AS1411 obtains its bioactivity as a cancer therapy, most likely due to its unique quadruplex structure when in solution [28]. AS1411 acts as an aptamer that binds nucleolin, a protein found on the surface and in malignant cells’ cytoplasm but absent from most normal cells’ surface and cytoplasm. Nucleolin is over-expressed in GBM and is a key regulator of proliferation and survival of GBM and thus is considered a great target for generating new therapies [29,30,31]. AS1411’s nucleolin targeting ability has been proven safe and non-toxic through phase I and II clinical trials [24,32,33,34]. Moreso, AS1411 has been used in gold nanoparticle systems to increase its bioactivity in many cancers and diseases [34,35,36,37].

Colloidal gold has been used for more than a thousand years for medicinal purposes [4]. Gold nanoparticles have been used more recently (since 1971) since Faulk and Taylor [38] first described antibody conjugation to gold. Since then, multiple biomolecules have been used to functionalize gold nanoparticle surfaces for genomics, biosensors, bioimaging, and targeted delivery of drugs, DNA, and antigens for therapeutic use [39]. Nanocarriers are a preferred method of delivery over normal therapeutic delivery due to good advantages including (1) an increased surface-to-volume ratio allowing for rapid release of drugs to targeted areas [40], (2) tunable surface chemistries which allow for a diverse range of therapies to be delivered, (3) reduction of therapy dose required, making a nanotherapy designs more cost-effective and (4) the ability to alter the size of nanocarriers giving the ability to administer drugs through many routes [41,42]. Gold nanoparticle (GNP) surfaces can be easily modified through the conjugation of molecules containing thiol (SH) groups utilizing highly predictable and well-characterized chemistries [42,43,44]. Oligonucleotide therapies, such as AS1411, can be superior conjugated ligands to GNPs due to their biological relevance, high specificity, selectivity, and versatility in conjunction with their easy chemical modification [44,45]. Additionally, multiple molecules can be conjugated to GNP surfaces to support the effective delivery of drug therapies by conferring in vivo stability, such as polyethylene glycol (PEG) [46].

Certainly, AS1411 and GNP designs have contributed to generating new and novel therapies for various cancers [14,34,37,47,48,49,50,51,52,53,54]. However, current AS1411-mediated drug delivery primarily focuses on using AS1411’s nucleolin targeting as a standalone therapy or on delivering chemotherapy agents. Little research has been done to functionalize GNPs with a base conjugation of AS1411 while allowing for additional GNP modification to provide non-chemotherapy agents. Because of this and the rising need for generating new successful and non-invasive GBM therapies, GNPs conjugated with AS1411 molecules hold potential for applications in the delivery of non-chemotherapy GBM therapies. An emerging class of oligonucleotide therapies, spherical nucleic acids, further supports this. They have shown the ability to surpass the blood–brain barrier—a significant consideration when creating GBM-specific treatments [55,56,57]. Given this, we have aimed to develop an optimal AS1411/GNP system tailored to act against GBM. Ideally, an optimal system against GBM would allow for the conjugation of bioactive molecules to GNPs to have a maximum anti-GBM response such as (1) affect hallmarks of tumorigenesis as indicated by a decrease in GBM metabolic activity, proliferation, and invasiveness and (2) have broad changes in GBM in vitro morphology.

## 2. Materials and Methods

### 2.1. Materials

HAuCl_4_·3H_2_O was purchased from Alfa Aesar (Tewksbury, MA, USA). Citric acid, trisodium salt (Na_3_C_6_H_5_O_7_), sodium borohydride (NaBH_4_), dithiothreitol (DTT), and anhydrous sodium bicarbonate (NaHCO_3_) were purchased from Sigma Aldrich (St. Louis, MO, USA). Nanopure ultrapure water (Barnstead, resistivity of 18.2 MΩ-cm) was used for preparing all aqueous solutions. 10.0X phosphate-buffered solution (pH 7.4) was purchased from Thermo Fisher Scientific (Waltham, MA, USA), used for salting particles, and used for subsequent dilutions where 1X PBS is required. Hydrochloric acid (HCl) and nitric acid (HNO_3_) were analytical grades and purchased from VWR (Rednor, PA, USA). Aqua regia solution (3 parts HCl and 1 part HNO_3_), was used to clean all glassware for GNP synthesis. Thiol Polyethylene Glycol-4-alcohol (SH-PEG-OH; Molecular Weight 210.3 g/mol; 95% purity) was purchased from BroadPharm (San Diego, CA, USA) and prepared for use via company specifications. Oligonucleotides having a regular DNA backbone (phosphodiester), a 5′-Thiol C6 S-S modification (Thio-MC6-D), 5′-6T spacer (for AS1411 and CRO), and high-performance liquid chromatography purification were supplied by Integrated DNA Technologies (Coralville, IA, USA). The oligonucleotide sequences used (including 6-T spacer) were 5′TTTTTTGGTGGTGGTGGTTGTGGTGGTGGTGGTTT (AS1411) and 5′-TTTTTTCCTCCTCCTCCTTCTCCTCCTCCTCCTTT (CRO). Fluorophore-labeled oligonucleotides (Cy5-AS1411 and Cy5-CRO) are identical in sequence to non-labeled versions with the addition of a 3′ modified fluorescent Cyanine-5 (Cy5) and also obtained from Integrated DNA Technologies. Illustra NAP-25 DNA size exclusion chromatography gravity columns were acquired from GE Healthcare Life Sciences (Pittsburgh, PA, USA). Amicon Ultra 15.0 mL centrifugal filters with Ultracel-30 (30,000 MWCO) were purchased from Merck Millipore (Billerica, MA, USA). UV absorption spectra of nanoparticle formulations and oligos were measured with a UV Visible Spectrometer (Varian Cary 50 BIO UV, Agilent Technologies, Santa Clara, CA, USA). Dynamic light scattering and zeta potential measurements were acquired on nanoparticle formulations using a NanoBrook Zeta PALS Zeta Potential Analyzer (Brookhaven Instruments, Holtsville, NY, USA). U87MG glioblastoma cancer cells were purchased from ATCC (Manassas, VA, USA). Dulbecco’s Modified Eagle Medium, Heat Inactivated Fetal Bovine Serum, and 10X Trypsin were purchased from Thermo Fisher Scientific. 100X Penicillin/Streptomycin mixture (Marlborough, MA, USA) was purchased from Cytiva. Glioblastoma cells were subcultured in T25 or T75 sterile culture plates from Corning Incorporated (Tewksbury, MA, USA). For experimental studies, U87MGs were passaged into sterile culture plates from VWR (Radnor, PA, USA) for Brightfield microscopy and metabolic studies or in 10 mm glass bottom, poly-d-lysine coated Matek dishes purchased from MaTek Life Sciences (Ashland, MA, USA) for confocal studies. Metabolic activity on nanoparticle or control-treated U87MGs was measured using (2,3-Bis-(Methoxy-4-Nitro-5-Sulfophenyl-2H-Tetrazolium-5-Carboxanilide) (XTT) acquired from Biotum (Fremont, CA, USA).

### 2.2. Co-Conjugated PEG and AS1411 GNP Synthesis

4 nanometer (nm) citrate-capped GNPs were sterilely synthesized at room temperature using previously reported protocols [58,59]. Briefly, 95 mL of 18.2 MΩ-cm nanopure ultrapure water was mixed with 2.5 mL of 0.01 M Na_3_C_6_H_5_O_7_. Then, 2.5 mL of 0.01 M HAuCl4 was added, mixed for 18 s, and reduced with 3 mL of 0.1 M NaBH_4_. Bare GNPs were stirred for 2 h before further modifications. Thiol-modified AS1411 (SH-AS1411) contains disulfide linkages upon purchasing for storage and stability that were cleaved before conjugation. This is done via boiling the required amount of SH-AS1411 necessary for conjugation, 250 µL of 1.0 M DTT, 1800 µL of 0.25 M phosphate buffer (PB), and enough nanopure ultrapure water to complete a boiling volume of 2.5 mL for 1 h followed by cooling for another hour. SH-AS1411 was isolated via size exclusion chromatography using Illustra NAP-25 DNA gravity columns and 0.1 M PB as the eluent. Gravity columns were primed with 25 mL of eluent before AS1411 isolation. The entire 2.5 mL of annealed AS1411/DTT mixture was eluted through the columns and collected in 3.5 mL of pure 0.1 M PB. SH-PEG alcohol was diluted in DMSO according to manufacturer specifications. Cleaved and purified SH-AS1411 and SH-PEG alcohol were then simultaneously conjugated to GNPs in different ratios (see next paragraph), generating a two-component PEG/AS1411 coating. Stepwise addition of 10X phosphate-buffered saline (PBS) over 96 h up to a concentration of 1X (based on final volume of particles synthesized before salting) followed by maximum sonication in a water bath for 10 min. Centrifugation at 13,500× *g* for 20 min, followed by a triplicate 1X-PBS washing and re-centrifugation after each wash removed any non-conjugated components.

Multiple PEG/AS1411 co-conjugated GNP formulations were synthesized that differed by their loading ratios of PEG and AS1411. Maximum possible loading onto GNPs was held constant at 12 times (expressed as 12X) the concentration of gold nanoparticles present within the colloidal solution, as measured by ultraviolet-visible (UV-VIS) spectrometry [41]. This 12X loading was divided up into multiple ratios of PEG:AS1411 and resulted in the following experimental conditions preserved throughout this paper: no PEG loading with maximum AS1411 loading (12X), maximum PEG loading with no AS1411 loading (0X), and three different loading ratios of AS1411:PEG (3X, 6X, and 9X). For formulations below the maximum of 12X AS1411, PEG was introduced to fulfill the 12X maximum loading requirements. Co-conjugated GNPs bearing SH-PEG and a CRO sequence were synthesized similarly once an optimal loading ratio was determined.

### 2.3. Nanoparticle Characterization

Particle size (measured in nanometers), zeta potential (measured in millivolts), and polydispersity indices (unitless) were calculated for all nanoparticle types. Changes in absorption spectra recorded on a Varian Cary 50 BIO UV (Agilent Technologies, Santa Clara, CA, USA) spectrometer verified conjugations. Oligo loading was evaluated for each GNP formulation via a 72-h cleavage of oligos from GNPs by treatment in a mixture of 1X PBS with 1.0 M DTT followed by UV-VIS measurement of cleaved and purified oligos (via Illustra NAP-25 DNA gravity columns with 0.1 M phosphate buffer as the eluent). Moles of oligo were calculated from UV measurements using Beer’s Law and compared to moles of gold present in colloidal solution to determine an average number of oligos per GNP. Transmission electron microscopy (TEM) studies was performed on the FEI Tecnai F20 TEM to determine GNP morphology and distribution. A field emission gun (FEG) was used for the electron source and the studies were performed with an accelerating voltage of 360 keV.

### 2.4. Cell Culture

U87MGs were cultured in Dulbecco’s Modified Eagle’s Medium (DMEM) with 10% heat-inactivated fetal bovine serum and 1% penicillin/streptomyosin mixture (final concentrations of 100 units per milliliter and 100 micrograms per milliliter, respectively). All subcultures were passaged using 0.25% Trypsin and seeded at a minimum density of 5000 cells/cm^2^. U87MGs were seeded at densities representing 1000 cells/well onto sterile, clear polystyrene 96 well plates. U87MGs seeded onto plates or dishes were cultured for two days before GNP treatments to allow cells to acclimate to culture conditions.

### 2.5. Cytotoxicity and Specificity Studies

Cell and control wells in 96 well plates were incubated with nanoparticle formulations combined with DMEM ranging from 0–5 micromolar (µM) AS1411 for 72 h with no media changes. Control cells were treated with 10 µM AS1411, bare GNPs representing the highest gold concentration, or no treatment. XTT absorbance data was obtained from a Molecular Devices SpectraMax M2 Spectrometer running SoftMaxPro 7.0 software. GNP absorbance interference with the assay was corrected by treating wells with no cells with GNP treatments. The resulting XTT absorbances within the cell plates were subtracted from corresponding wells with cells and GNP treatments. Statistically significant cytotoxicity of GNP formulations on U87MGs was determined via two-way ANOVA analyses. The specificity of AS1411 was verified by comparing the cytotoxicity of optimally determined co-conjugated GNPs bearing AS1411 to those bearing CRO. IC_50_ values (or the concentration that effectively inhibits 50% survival) of co-conjugated GNPs were obtained using GraphPad Prism version 7.0.0 for Windows, GraphPad Software (San Diego, CA, USA; www.graphpad.com, accessed on 17 February 2022).

### 2.6. Microscopy Studies

10X brightfield images were acquired in 96 well plates using a Nikon TE200 Epiflorescent microscope (Melville, NY, USA) with a Coolsnap HQ CCD camera (Roper, Duluth, GA, USA) enabled with NIS Elements software on U87MGs treated with GNP formulations and controls 72 h before XTT treatments.

### 2.7. Proliferation Analysis

Cell and control wells in 6 well plates were incubated with optimal co-conjugated PEG-AS1411 GNPs combined with DMEM ranging from 0–5 micromolar (µM) AS1411 for 72 h with no media changes. Control cells were treated with 10 µM AS141 or no treatment. Cell counts were obtained by collecting the cells after the 72 h incubation period by washing plates with 1X PBS and treating them with Trypsin for 5 min. Manual counting using a hemocytometer was completed to acquire cell numbers and then compared to the initial seeding density of 90,000 cells/well. Statistically significant differences in cellular proliferation of U87MGs were determined via one-way ANOVA analyses.

### 2.8. Statistical Analysis

All data were collected and processed in Microsoft Excel. Appropriate statistical tests were completed using GraphPad Prism using a significance level of α = 0.05. Data are presented as mean values +/− standard deviation. Statistical tests reported are one or two-way ANOVAs with Bonferroni post hoc tests. Sample sizes are described where needed.

## 3. Results and Discussion

**Figure 1:**
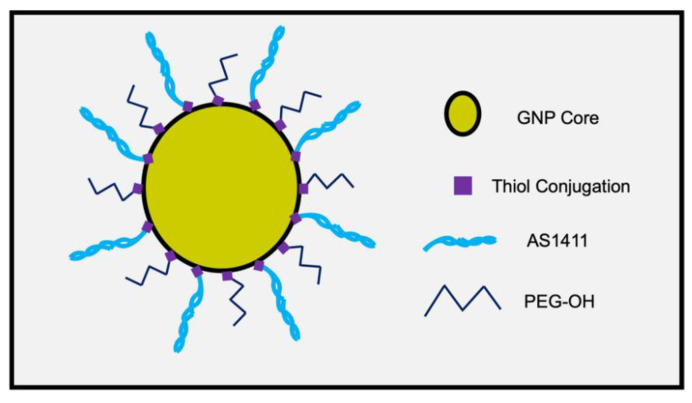
Schematic of PEG-AS1411 co-conjugated 4 nm GNPs.

**Figure 2:**
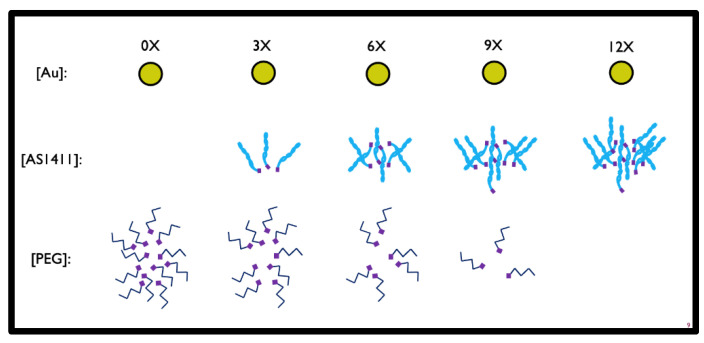
Visual description of GNP ratios tested.

To determine the optimal configuration of PEG-AS1411-GNP for GBM applications, human GBM-representing cell lines (U87MGs) were exposed to GNPs with differing loading ratios of PEG and AS1411 components. A schematic of the GNPs (Figure 1) shows the proposed topographic features. Multiple PEG/AS1411 co-conjugated GNP formulations were synthesized that differed by their loading ratios of PEG and AS1411 (Figure 2).

PEG is an anti-fouling molecule, thus protecting particles from protein aggregation, and is used as a surface modifier to enable further modification of GNPs with additional non-chemotherapy and anti-GBM therapies. Maximum possible loading onto GNPs was held constant at 12 times (expressed as 12X) the concentration of gold present within a colloidal solution, as measured by UV-VIS spectrometry. This 12X loading was divided up into multiple ratios of PEG:AS1411 resulting in the following experimental conditions, which were preserved throughout the experiments: no PEG loading with maximum AS1411 loading (12X), maximum PEG loading with no AS1411 loading (0X), and three different loading ratios of AS1411:PEG (3X, 6X, and 9X). For formulations below the maximum of 12X AS1411, PEG was introduced to fulfill the maximum loading requirements, maintaining the maximum 12X conjugation for all formulations.

### 3.1. Determining Optimal Synthesis of PEG-AS1411 Co-Conjugated GNPs

#### 3.1.1. Synthesis of PEG-AS1411 Co-Conjugated GNPs

**Figure 3:**
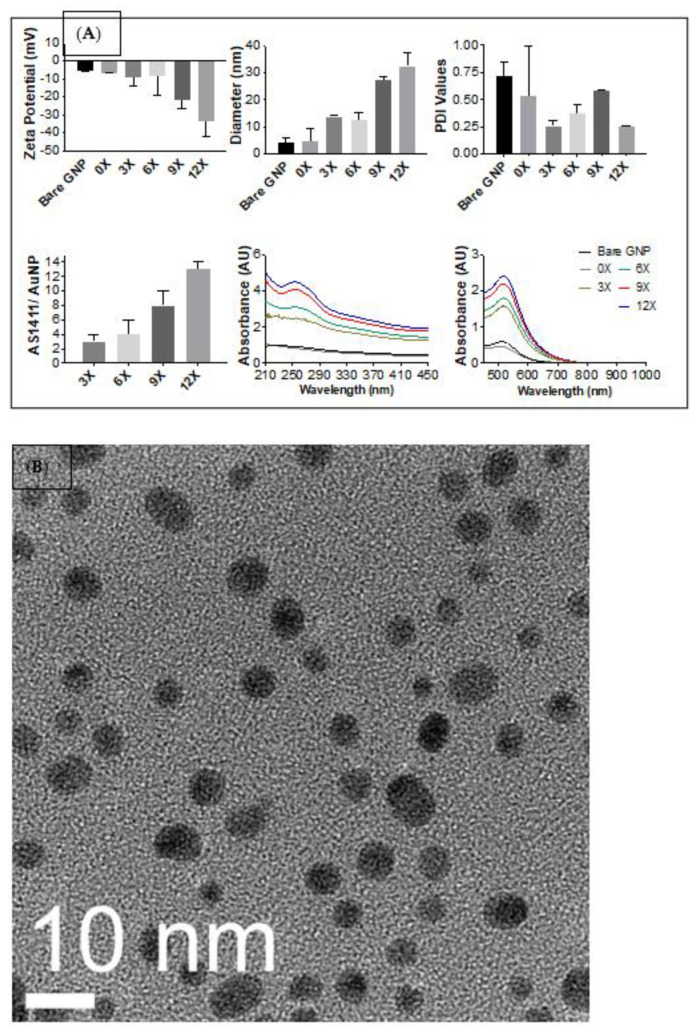
(**A**) Characteristics of various syntheses of PEG-AS1411 co-conjugated 4 nm GNPs. The top panel shows each synthesis’s zeta potential, diameter, and PDI values and compares them to citrate-capped bare GNPs. The bottom panel shows oligo loading within particle syntheses and resulting UV-VIS spectra for each. (**B**) TEM image of GNP-AS1411 (0X). Average particle size was measured to be 4.53 ± 0.78 nm.

The successful synthesis of AS1411-PEG co-conjugated GNPs was confirmed using zeta potential (mV), hydrodynamic diameter (nm), polydispersity indices (unitless), oligonucleotide number per GNP, and UV/visible spectroscopy as shown in Figure 3A. The top panel shows zeta potential, hydrodynamic diameter, and polydispersity indices for each particle synthesis. Zeta potential measurements help identify surface charges of nanoparticle syntheses and are used as a guideline for implying particle stability [60]. Generally, an increase in magnitude suggests a more stable particle synthesis allowing for the proper interaction between particles and biological molecules conjugated onto the surface. A gradual increase in zeta potential measurements is seen for all syntheses as AS1411 loading is increased. As designed, this loading is verified in the bottom panel of the figure, showing an increase in AS1411 loading across each synthesis. Benchmark values looked for from zeta potential measurements are usually any of those that are >20 mV in magnitude [61,62,63]. This would imply that 9X and 12X syntheses are the most stable, with zeta potential measurements reading −21.7 ± 4.57 mV and −33.5 ± 8.85 mV, respectively.

Hydrodynamic size measures the size of the gold core, conjugated biomolecules, and the hydration shell present around a particle when in solution. Generally, the hydrodynamic size will increase as biomolecules are conjugated to a surface. The reported size is used as a gauge to verify the conjugation of molecules and infer particle syntheses’ stability. A stepwise increase in hydrodynamic size is seen between pairs for all particle syntheses. 0X syntheses are most similar to bare citrate capped 4 nm GNPs. This is expected due to the size of the conjugated PEG molecules. 3X and 6X syntheses are relatively identical, as are 9X and 12X syntheses, while larger than 3X and 6X syntheses. This would indicate an effect on the size based on the distribution of the loading of AS1411 to the gold surface. This implies that the difference in loading between 3X versus 6X and 9X versus 12X is not as substantial as between 6X and 9X syntheses. It is believed that a saturation point is reached that is most prominently demonstrated in the 9X synthesis. This behavior is also seen in the UV-VIS spectra within the bottom panel of Figure 3. Absorption spectra at 520 nm exhibit an increase in absorbent units as the particle size increase with the spectra of bare GNPs and 0X, 3X and 6X, and 9X and 12X behaving similarly within their pairs. Within these couplet groups, bare GNPs (4.3 ± 1.4 nm), 3X (13.7 ± 0.4 nm), and 9X (27.4 ± 1.3 nm) syntheses are considered more stable due to the size of their variation. Absorbance maxima at 520 nm wavelengths additionally show a slight shift to the right, suggesting a successful surface conjugation [64]. Further demonstration of particle sizes and topography were confirmed via TEM imaging, Figure 3B.

Polydispersity indices measure the distribution of the recorded sizes within synthesis and are used as a gauge for particle stability and to state whether or not a synthesis is monodispersed (uniform with PDI values close to 0) or polydispersed (non-uniform with PDI values close to 1). Benchmark values of PDI are usually acceptable in the range of 0.5 to 0.7 [61,62,63]. Once AS1411 is conjugated to the surface of the GNP, our PDI values lower, indicating an increase in particle uniformity due to the oligonucleotide loading. All particle syntheses indicate good PDI values. Together with zeta potential and hydrodynamic size results, we can see an optimal particle synthesis among the 9X or 12X synthesis suggested by the nanoparticle characterization.

#### 3.1.2. Cytotoxicity of PEG-AS1411 Co-Conjugated GNPs

**Figure 4:**
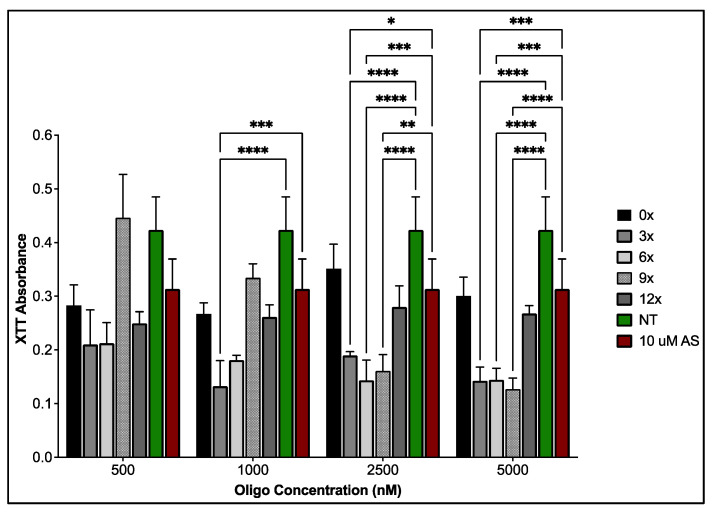
As measured by XTT assay, antiproliferative activity within U87MGs post-treatment with various PEG-AS1411 co-conjugated 4 nm GNPs. The legend shows particular particle syntheses for each concentration of oligonucleotide (AS1411) present within GNP syntheses. Groups treated with 0X represent the maximum amount of gold present within each concentration. NT represents non-treated control groups (media only). * represents *p* < 0.05, ** represents *p* < 0.01, *** represents *p* < 0.001, **** represents *p* < 0.0001.

**Figure 5:**
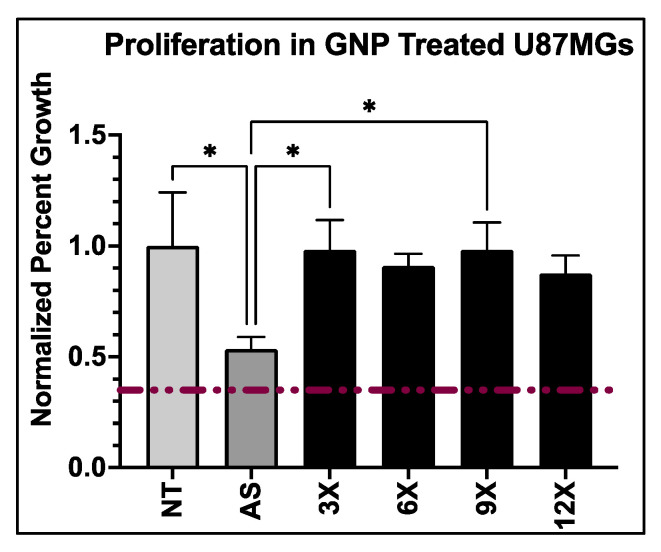
Proliferation activity, measured by cell viability, within U87MGs post-treatment with bare 4 nm GNPs representing maximal gold concentrations present within the solution for each particle type. * represents *p* < 0.05. The red line indicates the normalized initial cell seeding value.

To aid in determining an optimal PEG-AS1411 co-conjugated 4 nm GNPs for glioblastoma therapy, their in vitro anti-proliferative activity within U87MG cells was determined. Nanoparticle effects on GBM’s antiproliferative activity were determined using XTT assays measuring cellular metabolism (Figure 4). Unsurprisingly, 0X syntheses showed a minimal effect on antiproliferative activity due to the lack of any conjugated AS1411 to the GNP surface. This also indicates the relative safety of the conjugated PEG molecule and implies there is no inherent profound toxicity to GBM from this specific PEG on 4 nm GNPs. Interestingly, 3X, 6X, and 12X syntheses behaved similarly with acute toxicity at lower concentrations of AS1411 but had no cytotoxic profile as concentrations of AS1411 increased, with 12X syntheses having less of an overall effect on antiproliferative activity. 9X syntheses presented with the most standard cytotoxic profile and gradually increased antiproliferative activity as AS1411 concentration increased. 9X GNP syntheses significantly increase antiproliferative activity in GBM cells when compared to no treatment and 10 micromolar (µM) AS1411 control groups at 2.5 and 5 µM (62.05% and 48.69% decreases in XTT measurements with *p* < 0.0001 and *p* < 0.0018, respectively, at 2.5 µM—70.07% and 59.53% decreases in XTT measurements with *p* < 0.0001 for both comparisons at 5 µM). Although 3X syntheses presented with an early significant decline in activity, the lack of a cytotoxic profile, and other behavior on cellular morphology, discussed next, ruled them out as a promising candidate as the optimal particle type. Lastly, these effects are unlikely to result from the different gold concentrations introduced among particle treatments. Proliferation assays completed on U87MGs with bare GNPs representing maximal gold content (present in 5 µM groups; Figure 5) show no significant increase in antiproliferative activity for each particle type. Therefore, any concentration present at lower amounts should not have an effect either.

It is known that the surface coverage of thiol-bearing groups reaches saturation at approximately 75% coverage of the gold surface [65]. For our purposes, oligonucleotide loading of 4 nm GNPs at 12X oligo concentration is regarded as 100% coverage of the 4 nm surface. This parameter is based on max loading calculations comparing 4 nm GNP surface area (approximately 50.2 nm^2^) to the estimated surface area taken up by a molecule AS1411 in its quadruplex state at the point of thiol-GNP surface contact (4.15 nm^2^). Suppose saturation is measured by the bioactivity of AS1411, a known anti-cancer agent. In that case, this saturation fact is supported here since it was determined that the 9X syntheses (75% of the theoretical max) possess the most optimal bioactivity against GBM compared to other syntheses. The 9X syntheses are most likely providing the necessary environment for AS1411 to maintain its anti-cancer activity biostructure while simultaneously not interfering sterically from too much or not enough loading onto the GNP surface.

**Figure 6:**
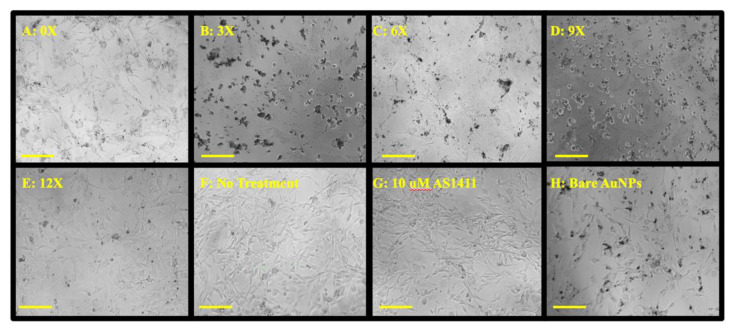
Morphological effects on U87MGs from treatment with co-conjugated PEG-AS1411 4 nm GNPs. Panels (**A**–**E**) represent 1 µM treatment groups. Panels (**F**–**H**) represent control groups. The scale bar indicates 100 microns.

Due to a litany of reasons as to why one sees cellular effects from an anticancer treatment, and given that any in-depth analysis of such processes with multiple particle types would be time-consuming, expensive, and inefficient, we examined the effects of our particle treatments on GBM cellular morphology (Figure 6) to help determine an optimal particle formulation that exhibits clear and profound cellular effects that can be used for additional in-depth cellular analyses. Screening the particles to see any morphological effects further suggests that XTT data from 9X particles may act as an optimal GNP synthesis for anti-GBM applications. GBM morphology in the 9X panel (Figure 6D) compared to the non-treated panel (Figure 6F) shows an apparent alteration to the classical neuronal morphology. GBM cells became more circular and spread out, affecting cellular function. While bare gold expresses no significant effect on U87MG anti-proliferative ability (Figure 5), Figure 6H shows an apparent effect on cell density, suggesting that bare GNPs affect normal cell behavior but not at cytotoxic levels. This result has also been reported when investigating the effects of bare gold interactions with cells [66,67,68]. Additionally, the 9X treatment (Figure 6D) lacks the intense black spots due to nanoparticle aggregation found in the 3X, 6X, and bare GNP treatments,—indicating greater nanoparticle stability under the treatment conditions for the 9X nanoparticles. Lastly, this panel is most closely related to cell morphologies of cells undergoing methuosis, a non-apoptotic cellular death pathway largely implicated in AS1411-based treatments [69,70,71]. Thus, it is assumed that because of the similarities in cellular morphologies reported in the literature [28,72], AS1411 could be the most bioactive in the 9X syntheses. Taken together with XTT and characteristic data, the 9X formulation was chosen as the optimal formulation in that it (1) affects hallmarks of tumorigenesis as indicated by a decrease in GBM metabolic activity, (2) induces broad changes in GBM in vitro morphology, and (3) maintains reported AS1411 bioactivity.

### 3.2. Specificity of Optimal PEG-AS1411 Co-Conjugated GNPs

**Figure 7:**
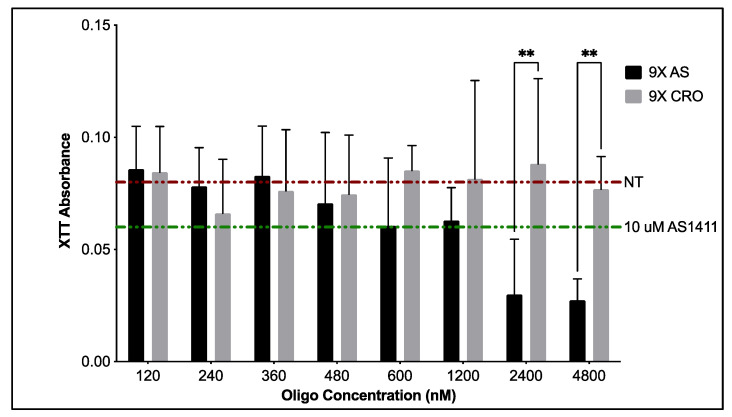
As measured by XTT assay, antiproliferative activity within U87MGs post-treatment with optimal 9X PEG-CRO control or PEG-AS1411 co-conjugated 4 nm GNPs. A significance level of ** represents *p* < 0.01.

To verify that the effects seen from 9X GNPs are due to the anti-cancer aptamer AS1411, optimal GNP syntheses were generated with a control oligonucleotide, CRO, where each guanine base in AS1411 is replaced with cytosine. Unlike AS1411, the CRO sequence is not known to form quadruplex structures. XTT metabolic assays compared the bioactivity of 9X PEG-AS1411 4 nm GNPs to that of 9X PEG-CRO 4 nm GNPs (Figure 7). From this data, we can see the dose-dependent response on the decrease of antiproliferative activity from 9X GNPs bearing AS1411, ultimately leading to a 75.0% decrease in metabolic activity at concentrations close to 5 µM oligo when compared to the non-treated control. GNPs bearing CRO oligos cause only a 5% decrease in antiproliferative activity for U87MGs compared to the non-treated control. This value remains consistent for most concentrations tested with GNPs bearing CRO oligos. 9X 4 nm GNPs bearing AS1411 oligo begin to show significant effects on U87MG metabolic activity when compared to optimal 9X 4 nm GNPs bearing CRO oligo at higher concentrations of oligo (~2.5 and 5 µM), indicating their specificity towards anti-GBM like activity (*p* < 0.0012 and *p* < 0.0017, respectively). Further studies using non-cancerous, brain-related cells such as neural progenitor cells can confirm the specificity of AS1411-coated GNPs.

### 3.3. Anti-GBM Activity of PEG-AS1411 Optimal Co-Conjugated GNPs

**Figure 8:**
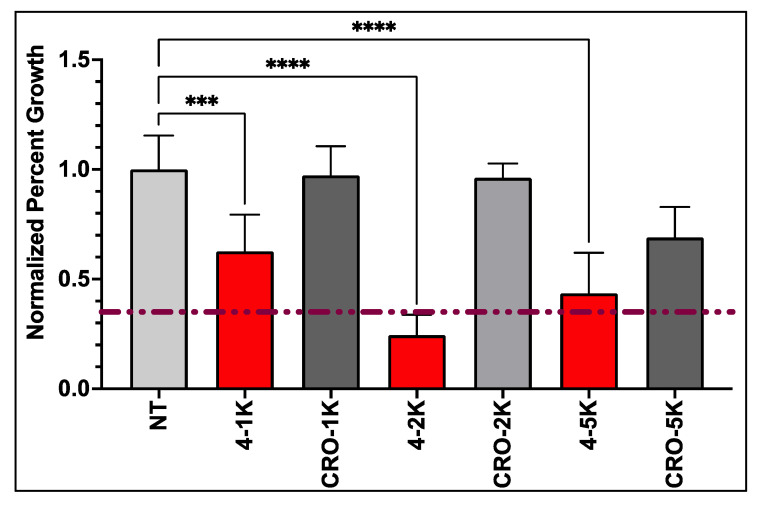
Proliferative effects at differing oligo concentrations on U87MGs from optimal 9X co-conjugated 4 nm GNPs conjugated with either PEG/AS1411 or PEG/CRO. Statistical significance of *** represents *p* < 0.0002 and **** represents *p* < 0.0001. No treatment (NT) and 10 µM AS1411 treatments serve as controls. Percent growth measurements normalized to initial seeding density (dashed red line).

Previous metabolic assays reported here can only be used as a screening tool, do not offer any insights into the mechanism of action of PEG-AS1411 co-conjugated GNPs, and have interference by GNPs in evaluating results. Thus, assays measuring specific hallmarks of GBM more directly and without potential interference from GNPs are preferred. Proliferation is a hallmark of GBM, contributing to its highly invasive and infiltrative nature and clinical progression. Several cellular signaling pathways are altered within GBM to create an environment of uncontrollable growth. Therefore, to determine if optimal PEG-AS1411 4 nm GNPs could be a specific and advantageous anti-GBM therapy, their effects on GBM cell proliferation were examined. Any effects on GBM proliferation from the treatment of 9X 4 nm GNPs with PEG and AS1411 can be used as a gauge to further define their bioactivity against GBM. From the data (Figure 8), we can see that proliferation of U87MGs is decreased in a dose-dependent manner with the treatment of PEG-AS1411 co-conjugated 4 nm GNPs at 1 µM, 2 µM, and 5 µM causing a significant decrease in proliferation by 75.57% and 56.49% (*p* < 0.0001 for both) for both 2 µM and 5 µM treatments, respectively. Additionally, 4 nm GNPs bearing control oligo (CRO) treated at the same concentrations of oligonucleotide show no significant effect on the proliferation. These data imply that our optimal GNP therapy reported here is specific to GBM and effective at altering GBM proliferation, significantly contributing to GBM aggression. This effect is most likely due to AS1411′s mode of action through its interaction with its protein target, nucleolin (NCL) which has recently gained interest as a biomarker of cellular proliferation [37,73]. Mechanistic studies in other cancer-representing cells have aimed at elucidating AS1411′s antiproliferative role. These have shown that treatment with AS1411 modestly increases EGFR phosphorylation leading to an increase in phosphorylation of Akt and Rac1 proteins, which most likely contributes to its anti-proliferative nature [69]. Analyzing gene expression of major cancer-related genes in cancerous cells has begun to elucidate additional cell cycle mediators (TP53, CDK proteins, MDM proteins, and BCL2/BAX) of this activity, but specific mechanisms are still unclear [35,74]. Other studies into effects on GBM regulation of proliferation using NCL interaction with AS1411 have also shown large decreases (upwards of 40% decrease) in U87MG proliferation after 48 h of 5 µM AS1411 treatment and upwards of 50% decrease after 72 h [73]. Here, we present improved effects (75% decreases) achieved at lower concentrations of AS1411 (2 µM). This confirms the increased bioactivity of AS1411 against its cancer targets once placed within a nanocarrier system, as seen in previous studies, with the antiproliferative effects consistent across two cancer cell types [34]. Ultimately, the optimized co-conjugated PEG-AS1411 4 nm GNP carrier system could be a promising therapy against GBM.

## 4. Conclusions

Here, we have presented the development and optimization of a PEG-AS1411 co-conjugated 4 nm GNP nanocarrier system designed to be optimally active against glioblastoma and allow for further modification with non-chemotherapy agents. Optimal 9X loaded GNPs were stable. Screening particle syntheses with various loading profiles further implicated 9X loaded syntheses as the optimal particle type producing typical cytotoxic profiles with the most notable effects on the metabolic activity of U87MGs and the most significant impact on cellular morphology—indicating alterations to normal U87MG cellular function. Optimal particles have pronounced uptake in the cellular environment after 72 h of treatment and retain the known specificity of AS1411. Most notably, optimal particles have a pronounced effect on U87MG proliferation. Due to the design of this therapy, and its impact on proliferation, it serves as a rationale for it to be used as a carrier to deliver further anti-GBM molecules. Further studies currently in progress are working to evaluate the anti-GBM performance of 9X particles, specifically looking at their effects on crucial regulator molecules (microRNA-21) and essential cellular proteins (PTEN, STAT3, PDCD4) affecting overall GBM proliferation, invasiveness, and survivability. Ongoing studies are looking at the effect in vivo performance of our optimal PEG-AS1411 co-conjugated 4 nm GNPs.

## 5. Patents

ANTI-NUCLEOLIN AGENT CONJUGATED NANOPARTICLES. Filed September 2019. Application No. PCT/US2020/050261.

## Data Availability

Restrictions apply to the availability of these data. Data was obtained from University of Louisville and are available from Martin O’Toole and Paula Bates with the permission of Qualigen Inc.
